# Dolutegravir for second-line treatment: Programmatic implications of new evidence

**DOI:** 10.4102/sajhivmed.v23i1.1428

**Published:** 2022-09-05

**Authors:** Ying Zhao, Gary Maartens, Graeme Meintjes

**Affiliations:** 1 Department of Medicine, Faculty of Health Science, University of Cape Town, Cape Town, South Africa; 2 Wellcome Centre for Infectious Diseases Research in Africa, Institute of Infectious Disease and Molecular Medicine, University of Cape Town, Cape Town, South Africa; 3 Division of Clinical Pharmacology, Department of Medicine, University of Cape Town, Cape Town, South Africa

Dolutegravir, an integrase strand transfer inhibitor, with an optimised nucleoside reverse transcriptase inhibitor (NRTI) backbone is the World Health Organization (WHO)-recommended second-line antiretroviral therapy (ART) regimen for adults after failing a first-line regimen based on a non-nucleoside reverse transcriptase inhibitor (NNRTI), either nevirapine or efavirenz.^1^ This WHO recommendation is based on the DAWNING study, which showed that dolutegravir was superior in both safety and efficacy compared to lopinavir-ritonavir, when administered with two NRTIs, at least one of which had to be fully active on resistance testing.^2^ The World Health Organization recommends substituting tenofovir with zidovudine when switching to second-line ART to ensure that there will be at least one fully active NRTI because the signature tenofovir resistance mutation K65R does not compromise zidovudine’s effectiveness and there is limited access to resistance testing in high-burden, resource-limited settings to select an optimised NRTI backbone.^3^

The question has been raised whether recycling tenofovir and lamivudine (or emtricitabine) with dolutegravir in second-line ART could be an effective and easily implementable regimen. Tenofovir is less toxic than zidovudine^4^ and is dosed once rather than twice daily, which improves adherence. Recent evidence has shown that recycling tenofovir in second-line ART is efficacious. The NADIA study randomly assigned participants in a 2 × 2 factorial design to daily dolutegravir or darunavir-ritonavir combined with either tenofovir or zidovudine (both with lamivudine).^5^ At week 96, recycling tenofovir was superior to switching to zidovudine (percentage point difference 7.0%, 95% confidence interval [CI]: 1.2% – 12.8%).^5^ ARTIST, a prospective cohort study of recycled tenofovir and lamivudine with dolutegravir in second-line ART, reported that 85% of 60 participants achieved HIV-1 RNA < 50 copies/mL at week 24, despite 65% having resistance to both tenofovir and lamivudine at baseline.^6^ In the VISEND randomised trial, 83% achieved HIV-1 RNA < 1000 copies/mL in the tenofovir-lamivudine-dolutegravir group at week 48, compared with 82% in the atazanavir-ritonavir-zidovudine-lamivudine group and 69% in the lopinavir-ritonavir-zidovudine-lamivudine group.^7^ It is well established that the modest effect of NRTIs on reducing viral fitness in the presence of NRTI resistance is both necessary and sufficient to achieve virologic suppression in combination with a protease inhibitor^8^ – this is likely also true for dolutegravir as over 90% of those taking either dolutegravir or darunavir-ritonavir and two NRTIs with resistance to both NRTIs achieved virologic suppression in the NADIA study.^5^ In our view, these findings from recent studies strengthen the evidence base for recycling tenofovir and lamivudine (or emtricitabine) with dolutegravir in second-line ART in resource-limited settings.

There is an important caveat for clinicians to be aware of: resistance to dolutegravir has been documented more frequently in second-line when compared with first-line ART. Emergent dolutegravir resistance was reported in a small proportion of integrase inhibitor-naïve participants switching to second-line dolutegravir-based ART in randomised trials (9 [4%] of 235 participants by week 96 in NADIA and 6 [2%] of 314 participants by week 159 in DAWNING).^2,5,9^ Notably, emergence of protease inhibitor resistance did not occur in either NADIA or DAWNING, indicating that dolutegravir has a lower genetic barrier to resistance than protease inhibitors when dolutegravir is administered with NRTIs potentially compromised by resistance mutations. Risk factors associated with emergent dolutegravir resistance include intermittent adherence, drug-drug interactions, high baseline HIV-1 RNA and active opportunistic infections.^9^ Further research is needed to better understand the risks associated with the development of dolutegravir resistance and particularly when combined with pre-existing resistance to NRTIs, as well as strategies to mitigate dolutegravir resistance selection and second-line failure.

Darunavir-ritonavir was non-inferior to dolutegravir for the outcome of virologic suppression at week 96 in the NADIA study^5^ and is, therefore, a robust alternative to dolutegravir in second-line ART. The cost and availability of a fixed-dose combination with NRTIs currently favour the use of dolutegravir over darunavir-ritonavir. Darunavir-ritonavir with two NRTIs, even if there is resistance to both these NRTIs, should be an effective treatment option if virologic failure with dolutegravir resistance develops on dolutegravir-based second-line ART.

The NADIA study investigators argue that patients switching to dolutegravir after virologic failure on a NNRTI-based first-line regimen are a high-risk group for developing resistance and HIV-1 RNA rebound on dolutegravir-based second-line regimens should trigger intensive adherence counselling and an earlier repeat HIV-1 RNA test following adherence interventions, based on the observation that most participants who developed dolutegravir resistance self-reported suboptimum adherence at multiple study visits.^5^ A cohort study in East and Central Africa reported that patients who switched from a first-line NNRTI regimen to dolutegravir with HIV-1 RNA ≥ 1000 copies/mL or unknown HIV-1 RNA levels had worse HIV treatment outcomes compared with those who switched with HIV-1 RNA < 200 copies/mL.^10^ Therefore, these patients may benefit from additional clinical monitoring and adherence support.

In summary, dolutegravir in second-line ART with recycled tenofovir is more effective than switching to zidovudine. However, emergent dolutegravir resistance in a small minority of participants raises a public health concern as dolutegravir is recommended in most patients requiring first-line ART. There is, therefore, a need for appropriate surveillance programmes to monitor the emergence of dolutegravir resistance in second-line ART.
